# Case of Pelvic Tumour

**Published:** 1838-08-15

**Authors:** J. C. Hall

**Affiliations:** Washington City


(To be continued.)

---

*Case of Pelvic Tumour, by* J. C. HALL, M. D., *of Washington.*

To Drs. Biddle and Clymer.

*Gentlemen*—The enclosed, if worthy of insertion in your valuable journal, is at your service, and its publication will be gratifying to myself and others. Any information, bearing on the subject, that the experience and reading of yourselves or correspondents may afford, will be gratefully received by

Your friend and ob't serv't,

J. C. HALL.

The following case, from the standing of its subject, and the obscurity and duration of the disease, attracted much public and professional attention.

Mr. J—— C——, aged about forty, habituated, from early life, to admirable moral and physical discipline, and enjoying excellent health, on the 4th of March, 1837, was exposed for a long time, in a standing posture, to damp and cold. Soon after, he was affected with a pain in the left hip, supposed to be rheumatic. All the ordinary remedies failing to relieve him, he visited the Medicinal Springs, and exercised on horseback, which aggravated his symptoms. About the 1st of January last, it was suspected to be a case of coxalgia. At that time, the patient came under my care. He was paler and thinner than usual; but none of the vital functions seemed impaired, or indicated the existence of a serious disease. He bore his weight, habitually, on the sound limb, which produced an apparent lengthening of the other. He walked with difficulty and pain. There was no flatness of the nates; but, on the contrary, over and above the trochanter, there was a smooth, firm swelling, without discolouration, but very painful on pressure. The hip-joint admitted of very free passive motion, with but slight increase of pain. Percussion on the knee, gradual but forcible pressure on the trochanter, so as to grind the articular surfaces together, occasioned little or no uneasiness. The thigh was slightly wasted, and its muscles much relaxed. Pains occasionally darted down the course of the sciatic nerve, and there was a circumscribed but very intense one over the patella and instep, which, from the suddenness of its onset and departure, was evidently sympathetic, and which the patient described, as differing altogether from the fixed and gnawing pain about the hip. I did not recognise the diagnostic symptoms of coxalgia, but confessed my ignorance of the nature of the disease. The treatment pursued was confinement to the horizontal position, leeches, gentle purging, the moxa, an issue, iodine, moderate diet, warm and anodyne fomentations. No permanent benefit resulted. The tumour increased greatly, occupying all the dorsum of the ilium, and presenting an appearance, such as would arise from a hypertrophy of all the gluteii muscles, bounded by the crista ilii above, and extending to the trochanters below. It was tense, very elastic, painful throughout, and possessed a feeling of fluctuation, which tantalized us with the idea that it contained fluid, and that it would shortly discharge its contents.

The greater part of its surface was smooth, shining, and scarcely discoloured, but near and beneath the anterior spinous process of the ilium, several lumps or tubercles were felt. The pain was increased, and, at night, was frequently agonizing. The appetite and sleep were much impaired, and, early in March, hectic, with rapid emaciation, was apparent. About the 1st of April, the tumour somewhat subsided, and we were flattered with the hope of a recovery; but, on examination, a swelling was discovered extending along the whole of the margin of the crista of the ilium, and Poupart's ligament. This continued to increase until it reached nearly to the false ribs, and extended obliquely downward to the iliac fossa of the opposite side, thrusting the bladder and rectum completely to the right, and elevating the iliac artery, so as to make it apparent to the touch and sight.

The displacement of the vein produced great enlargement of its external branches, with œdema of the limb and loins; while the stretching of the femoral nerve caused a most agonizing burning sensation.

Violent strangury also harrassed our afflicted patient, and the inguinal glands became inflamed and enlarged, but never suppurated. In the latter part of May, a dreadful pain was felt, about the sixth dorsal vertebra, and extending around the chest; and tumours, having all the external character of that of the hip, made their appearance on both sides of that portion of the spine; at the end of a week, this pain suddenly subsided, and it was then discovered, that all sensation and motion from the middle of the thorax downward were gone.

The bladder required the catheter, and the bowels were emptied with difficulty. At last, on the 26th of June, after being in a moribund condition for two weeks, and after having endured immense suffering with unequalled fortitude, Mr. C—— found, in death, a welcome relief.

The treatment is not given in detail, as it was ineffectual, and not based on any accurate knowledge of the disease. In fact, the indications were so obscure, that we could not permit ourselves to adopt any other than palliative measures. It may be well, however, to mention that hot stupes to the hip, veratria ointment to the knee and instep, with morphia and the tincture of conium, internally, were generally successful in abating pain and procuring sleep.

By the voluntary request of Mr. C——, an examination of the body was made, eighteen hours after death. The emaciation was excessive; sphacelus had occurred extensively in the back and heels. All the viscera in the abdomen were in a healthy state. The ureters were much enlarged, from the extension to which they had been subject. The peritoneum exhibited no signs of recent or old inflammation. An immense tumour occupied the whole fossa of the ilium, ascending nearly to the ribs, and descending into the pelvic basin, filling it almost entirely, raising the bladder, and pressing the rectum against the right linea ileo-pectinea, so as to obstruct the passage of the fæces.

On dissecting up the peritoneum, which was easily detached, and raising the skin and fascia over the hip, the external and internal tumours were found to be continuous and invested with a very slight capsule. On laying the tumour open, we discovered that not a vestige of the gluteii or iliacus internus muscles remained, but, in their place, a grayish, inodorous substance, of the appearance and consistence of brain, with, here and there, small masses of gelatinous and sanious fluid, without any blood or pus. Poupart's ligament, and the sacio-ischiatic, all the anterior portion of the ilium, the superior ramus of the os pubis, and the entire acetabulum, except its lower margin, were gone, and their place occupied by this homogenous brain-like substance. The bony structure had entirely vanished; no loose carious portions were seen, and the bone had been divided as smoothly as with a saw. The tubercles, or lumps, before mentioned, were doubtless, fragments of detached bone.

The capsular ligament was destroyed, and the ligamentum teres was rotten. The head of the bone, still in situ, and covered with its cartilage, was permeable to the knife, and its neck deeply eroded. Many of the mesenteric glands were slightly enlarged, but none of the lymphatic.

The spine was not examined, but I have no doubt, that the swelling along the dorsal vertebræ, was of the same nature as that of the hip, and had invaded the spinal canal, causing by its pressure on the nerves and marrow, in the first instance, the dreadful burning pain, and, ultimately, by compression, destroying sensation and motion in the parts below.

My experience does not enable me to classify, with precision, this malignant tumour. Its deceptive feeling of fluctuation, the nature of the pain, its cephaloid structure, its converting every tissue to its own nature, assimilate it to fungus hæmatodes. But, on the other hand, the exemption of the skin from disease, the absence of fungus, the non-existence of a reticulated structure with grumous blood, the soundness of the lymphatics and viscera, with the general good health of the patient, till the last three months of his life, fail to correspond with the usual circumstances attending that affection.

The morbid affection, denominated by the French surgeons encephaloid disease, has many points in common with the one here detailed; but the case of a lady, described by Professor Warren, in his Observations on Tumours, (page 83,) an example of which had never been seen by Drs. Post and Hosack, and only once by Dr. Physick, approached somewhat to its character.

Mr. C—— was attended by Drs. F. May, Sewall, and myself, and had the advantage of a single visit from Professors N. R. Smith and Mussey.

All admitted the difficulty of a diagnosis, and of course refrained from expressing a confident opinion.

J. C. Hall.

*Washington City*, August 6th, 1838.

